# P2Y12 Receptor Antagonist, Clopidogrel, Does Not Contribute to Risk of Osteoporotic Fractures in Stroke Patients

**DOI:** 10.3389/fphar.2017.00821

**Published:** 2017-11-14

**Authors:** Niklas R. Jørgensen, Peter Schwarz, Helle K. Iversen, Peter Vestergaard

**Affiliations:** ^1^Research Center for Ageing and Osteoporosis, Rigshospitalet, Copenhagen, Denmark; ^2^Department of Clinical Biochemistry, Rigshospitalet, Copenhagen, Denmark; ^3^Odense Patient Data Explorative Network, Odense University Hospital, Institute of Clinical Research, University of Southern Denmark, Odense, Denmark; ^4^Department of Endocrinology, Rigshospitalet, Copenhagen, Denmark; ^5^Faculty of Health Sciences, University of Copenhagen, Copenhagen, Denmark; ^6^Stroke Unit, Department of Neurology, Rigshospitalet, Copenhagen, Denmark; ^7^Departments of Clinical Medicine and Endocrinology, Aalborg University Hospital, Aalborg, Denmark

**Keywords:** stroke, antiplatelet therapy, osteoporosis, bone, fracture, P2Y12 receptor, clopidogrel

## Abstract

**Background:** Stroke is a leading cause of mortality and morbidity. It is associated with excessive bone loss and risk of fracture in stroke patients is high. The P2Y12R antagonist and platelet inhibitor, clopidogrel, is widely used for secondary prevention after a stroke. However, recent studies have shown that clopidogrel has negative effects on bone and that long-term clopidogrel use is associated with increased fracture risk. The purpose of the current study was therefore to investigate the association of clopidogrel treatment with risk of fractures in stroke and TIA patients.

**Methods:** The study was a cohort study including all subjects who were prescribed clopidogrel between 1996 and 2008 in Denmark (*n* = 77,503). Age- and gender matched controls (*n* = 232,510) were randomly selected from the background population. The study end-points were occurrence of stroke or TIA and occurrence of fracture. Clopidogrel use was primary exposure.

**Results:** Ischemic stroke increased risk of fracture by 50% while haemorrhagic stroke and TIA increased the risk by 30%. However, after adjusting for multiple confounders only patients with ischemic stroke and haemorrhagic stroke had increased fracture risk. Clopidogrel use was not associated with increased fracture risk in subjects with ischaemic stroke or TIA. In contrast, after adjusting for multiple confounders clopidogrel treatment was associated with a 10–35% reduced risk of fracture.

**Conclusion:** Patients with stroke have increased risk of osteoporotic fractures, but clopidogrel treatment does not increase fracture risk. In contrast, patients less adherent to the treatment have lower risk of fractures than non-users and patients with high adherence. However, based on the increased risk in stroke patients, clinicians should consider evaluation of bone status of these patients.

## Introduction

Stroke is one of the leading causes of mortality and morbidity worldwide. In the surviving stroke patient the increased functional dependence and cognitive decline often leads to immobilization. Nearly 80–90% of all strokes occur in people over the age of 65, adding to the risk of osteoporosis in these patients (Truelsen et al., 2006). Balance is often impaired after a stroke increasing the risk of falls and in combination with immobilization and advanced age stroke patients have multiple risk factors for fractures. After a hemiplegic stroke, changes in bone metabolism occur already during the week following the stroke. Serum markers of bone formation are low and serum calcium and markers of bone resorption are increased and negatively correlated to the level of mobilization (Sato et al., 2000). Also, a high number of the patients had vitamin D deficiency, as only 27% had sufficient serum levels of vitamin D (Sato et al., 2000). Stroke patients with hemiparesis develop a significant bone loss during the first year after the stroke, primarily in the affected side and mainly in the humerus (loss in bone mineral density of 17.4% after 1 year) and the proximal femur (loss of 12.2%) (Ramnemark et al., 1999). Excessive bone loss is a clear risk factor of osteoporosis and subsequently fractures. A recent meta-analysis that included 13 cohorts of stroke survivors found a 50% increased risk of hip fracture in the stroke patients compared to healthy men and women of the same age (Yuan et al., 2016). In a Swedish cohort of stroke patients, the risk of fracture was 4% 1 year post-stroke, 15% after 5 years and 24% after 10 years (Ramnemark et al., 1998). Fractures occurred primarily after falls (84% of fractures) and mainly affected the paretic side in patients with paresis (Ramnemark et al., 1998). At least in younger individuals, no difference in fracture rates was found between stroke and TIA patients (Brown et al., 2008). Thus, post-stroke fractures may result from disuse hemiosteoporosis, hypovitaminosis D, and an increased risk of falls.

Treatment with platelet aggregation inhibitors has become the gold standard for secondary prevention of recurrent non-cardioembolic strokes after the initial episode, both in patients with ischaemic stroke and TIA (Kernan et al., 2014). Life-long treatment with the thienopyridine, clopidogrel, has become one of the standard treatments in patients suffering a stroke (Kernan et al., 2014). Clopidogrel inhibits platelet aggregation by irreversibly binding to the P2Y12 receptor, previously called the “platelet receptor.”

Recent studies have shown that the P2Y12 receptor is expressed not only on platelets but also on bone cells. Clopidogrel inhibits the function of the bone forming osteoblasts (Syberg et al., 2012). Moreover, in a recent *in vivo* study mice treated with clopidogrel had reduced bone mass and -strength of approximately 20% (Syberg et al., 2012). Thus, clopidogrel induced severe osteoporosis in the animals. In addition, a large population-based register study including all patients having prescribed clopidogrel in Denmark between 1999 and 2008 confirmed the suspected negative effects of clopidogrel on bone, as the fracture risk was increased by 60% compared to non-clopidogrel users (Jørgensen et al., 2012). Thus, clopidogrel treatment has clear negative effects on bone.

As patients with ischaemic stroke and TIA are prescribed clopidogrel for the rest of their lives and as they are already susceptible to bone fractures, additional bone loss induced by clopidogrel treatment may therefore increase the risk even further. The aim of the present study was to evaluate the association between the platelet inhibitor clopidogrel and fracture incidence in stroke and TIA patients in a population-based nationwide case-control study.

## Materials and Methods

### Study Design

The study was designed as a cohort study. All subjects who were prescribed clopidogrel during the years 1996 to 2008 in Denmark were included as exposed subjects (*n* = 77,503), and for each exposed subject three subjects of the same age (same birth year) and gender were randomly selected from the background population as controls (*n* = 232,510). The non-users were selected and matched on age- and gender to the users through an intensity sampling technique. As it is a register-based study, no approval from the Ethics Committee is necessary and Ethics approval was therefore not sought.

### End-Points

The study end-points were occurrence of stroke or TIA and occurrence of any fracture succeeding the stroke or TIA (International Classification of Diseases (ICD) 10 codes: S02.0-S02.9, S07.0-S07.9, S12.0-S12.9, S22.0-S22.9, S32.0-S32.8, S42.0-S42.9, S52.0-S52.9, S62.0-S62.9, S72.0-S72.9, S82.0-S82.9, S92.0-S92.9), hip fracture (S72.0 and S72.1), forearm fracture (S52.5 and S52.6), and spine fracture (S12.0, S12.1, S12.2, S12.7, S12.8, S12.9, S22.0, S22.1, S32.0, S32.7, S32.8) between January 1^st^ 1996 and December 31st 2008. The vertebral fractures included were clinical fractures collected from hospital records of patients, who were referred to emergency rooms or other departments and were diagnosed with a vertebral fracture.

### Exposure Variables

Clopidogrel use was the primary exposure. Patterns of drug use were analyzed for the period from January 1st, 1996 to the date of fracture or corresponding dummy date among the non-users. Data were collected systematically and information on whether the drugs were used systematically or temporarily was included in the analyses of drug use via the Defined Daily Dose (DDD), using the dates of prescription. DDD is used according to the WHO definition, which is defined as the assumed average maintenance dose per day for a drug used for its main indication in adults. The other exposure variables were occurrence of (1) use of drugs known to be associated with fracture risk (corticosteroids), and (2) co-morbidities known to affect fracture risk (prior fracture Klotzbuecher et al., 2000) and alcoholism (Kanis et al., 2005). These factors were chosen as they were known to potentially affect fracture risk, and were regarded as important potential confounders in a setting where many variables besides the main factor may influence the risk of fractures (confounding by indication). To analyze for confounding from use of other cardiovascular drugs these were also included (Rejnmark et al., 2005, 2006a,b).

Due to the imbalances in confounders typical for observational studies, extensive confounder control was performed including all the unevenly distributed known confounders: Charlson index, income, living alone or not, spironolactone use, use of bronchodilator drugs (proxy for smoking), use of drugs for smoking cessation (proxy for smoking), prior fracture, alcoholism, systemic corticosteroids, statin use, ACE use, ACE+diuretics use, combined alpha plus betablocker use, other diuretics use, betablocker use, betablocker plus other drug combined, calcium channel blocker, thiazide diuretics, loop diuretics, dipyridamole use, and acetylsalicylic use. The variables were entered into the statistical analysis and analyses for interaction were performed.

Information on bodyweight or body mass index (BMI) was not available.

### Registers Used

The information on fracture occurrence, stroke and TIA as well as occurrence of other diseases, prior fractures, alcoholism came from two registers: (1) The National Hospital Discharge Register (Andersen et al., 1999), and (2) The Psychiatric Central Register (Munk-Jørgensen and Mortensen, 1997). These have been described in detail previously (Mosbech et al., 1995; Andersen et al., 1999; Vestergaard and Mosekilde, 2002; Jørgensen et al., 2012; Vestergaard et al., 2012; Grove et al., 2013).

The information on clopidogrel use came from The Danish Medicines Agency, who keeps a nationwide register of all drugs sold at pharmacies throughout the country from 1996 and onward (The National Pharmacological Database run by the Danish Medicines Agency^1^). Any drugs bought are registered with ATC code, dosage sold, and date of sale for the period January 1st 1996 to December 31st, 2008. Details about this have previously been published (Vestergaard et al., 2012).

It is possible to link these sources of information through the Central Person Register Number, which is a unique registration code given to every inhabitant, enabling registration on an individual basis.

The project was approved and controlled by the National Board of Health, the Danish Data Protection Agency, and the Directory board of the Psychiatric Central Register.

In Denmark almost all patients with fractures are managed in the hospital system (also including the emergency rooms) (Vestergaard et al., 2002), even fractures sustained abroad are registered upon return for insurance reasons. The capture of fractures is thus very high (Mosbech et al., 1995; Andersen et al., 1999). The validity of a fracture diagnosis is around 93% (Vestergaard and Mosekilde, 2002).

### Statistical Analyses

Mean and standard deviation were used as descriptive statistics. Crude and adjusted hazard ratios (HRs), and 95% confidence intervals were calculated. Cox proportional hazard regression models were used to analyze the time to fracture in exposed vs. non-exposed subjects. The proportional hazard assumption was checked through inspection of survival plots. In addition, in the Cox regression models there was also tested for age and gender interaction. In order to avoid immortal time bias, patients are excluded from further risk analysis after subsequent stroke/TIA events.

Analyses were performed using STATA 9.0 (STATA Corp., College Station, TX, United States) and IBM SPSS 19.0 (SPSS Inc.).

## Results

### Baseline Characteristics of Users and Non-users

Clopidogrel-treated and untreated controls were well matched for both gender and age and the baseline variables in the two groups are shown in **Table 1**. Not surprisingly, the fraction of individuals with prior cerebral incidents such as ischaemic and haemorrhagic stroke and TIA were significantly higher in the clopidogrel group than in the control group. Also, the fraction of individuals with other cardiovascular diseases (myocardial infarction, heart failure, atherosclerosis and angina pectoris) was significantly higher among clopidogrel-users than in non-users. Moreover, the proportion of subjects on prior statin, ACE inhibitors, beta blockers, diuretics and other anti-platelet inhibitors was higher amongst users than in controls (**Table 1**).

**Table 1:** Baseline characteristics of clopidogrel users and non-users.

Parameter	Clopidogrel users (*n* = 77,503)	Non-users (*n* = 232,510)	*P*
Age (years, mean ± SD)	65.7 ± 12.7	65.7 ± 12.7	–
Men (number, %)	50,118 (64.7%)	150,354 (64.7%)	–
Women (number, %)	27,385 (35.3%)	82,156 (35.3%)	
Prior fracture	16,730 (21.6%)	47,795 (20.6%)	<0.01
Prior diagnosis of alcoholism	2,469 (3.2%)	6,562 (2.8%)	<0.01
Prior acute myocardial infarction	45,365 (58.5%)	10,709 (4.6%)	<0.01
Prior angina pectoris	53,523 (69.1%)	21,428 (9.2%)	<0.01
Prior heart failure	11,243 (14.5%)	8,129 (3.5%)	<0.01
Prior peripheral atherosclerosis	7,356 (9.5%)	7,193 (3.1%)	<0.01
Prior cerebral atherosclerosis	2,784 (3.6%)	2,900 (1.2%)	<0.01
Prior diabetes	10,863 (14.0%)	11,894 (5.1%)	<0.01
Prior atrial fibrillation	8,036 (10.4%)	12,080 (5.2%)	<0.01
Prior systemic corticosteroids	20,050 (25.9%)	47,067 (20.2%)	<0.01
Prior use of statins	30,345 (39.2%)	26,735 (11.5%)	<0.01
Prior ACE/ARB use	38,658 (49.9%)	46,380 (19.9%)	<0.01
Prior ACE/ARB plus diuretics use	6,529 (8.4%)	13,129 (5.6%)	<0.01
Prior combined alpha/beta blocker	2,897 (3.7%)	3,187 (1.4%)	<0.01
Prior use of other diuretics	8,737 (11.3%)	16,926 (7.3%)	<0.01
Prior use of betablockers	36,288 (46.8%)	43,114 (18.5%)	<0.01
Prior use of betablocker combinations	562 (0.7%)	1,249 (0.5%)	<0.01
Prior use of calcium antagonists	27,953 (36.1%)	41,744 (18.0%)	<0.01
Prior thiazide diuretic use	24,895 (32.1%)	53,675 (23.1%)	<0.01
Prior loop diuretic use	18,105 (23.4%)	31,725 (13.6%)	<0.01
Prior use of dipyridamole	8,123 (10.5%)	7,331 (3.2%)	<0.01
Prior use of low dose ASA	44,015 (56.8%)	54,610 (23.5%)	<0.01
Charlson index	2.1 ± 1.9	0.8 ± 1.5	<0.01
Income in index year (Danish crowns)	209,223 ± 315,460	224,149 ± 227,691	<0.01
Living with someone	29,030 (37.5%)	88,901 (38.2%)	<0.01
Bronchodilator drugs	23,900 (30.8%)	54,703 (23.5%)	<0.01
Drugs for smoking cessation	1,969 (2.5%)	2,240 (1.0%)	<0.01
Spironolactone	11,292 (14.6%)	11,889 (5.1%)	<0.01
Prior haemorrhagic stroke	924 (1.2%)	2,004 (0.9%)	<0.01
Prior ischemic stroke	7,227 (9.3%)	6,077 (2.6%)	<0.01
Prior TIA	5,573 (7.2%)	5,366 (2.3%)	<0.01

*Figures are numbers and % or mean and standard deviation (SD). ACE: Angiotensin-converting enzyme inhibitor, ARB, angiotensin receptor blocker; ASA, acetylsalicylic acid*.

### Fracture Risk of Cerebral Ischemic Events

The risk of fracture following TIA, ischaemic stroke and haemorrhagic stroke was calculated. A clear association between all three types of cerebral ischemic events and the risk of fracture was detected (**Table 2**). Ischemic stroke increased the risk of any fracture by approximately 50% while haemorrhagic stroke increased the risk by approximately 30%. Surprisingly, TIA also increased the risk of fracture to the same extent as haemorrhagic stroke. Even after adjusting for multiple confounders, a 14% increased risk of any fracture was seen in patients with ischaemic stroke and 19% increased risk of hip fractures was seen in patients with haemorrhagic stroke (**Table 3**).

**Table 2:** The frequency and risk of any fracture are presented.

Crude relative risk	Any fracture	Hip fracture	Forearm fracture	Spine fracture
	IRR (95% CI)	IRR (95% CI)	IRR (95% CI)	IRR (95% CI)
Clopidogrel	1.05 (1.02–1.09)^∗∗^	1.00 (0.94–1.07)	1.03 (0.97–1.10)	1.13 (0.99–1.28)
TIA	1.28 (1.20–1.36)^∗∗∗^	1.50 (1.32–1.71)^∗^	1.18 (1.00–1.38)^∗^	1.34 (1.00–1.79)^∗^
Ischaemic stroke	1.54 (1.46–1.64)^∗∗∗^	1.55 (1.24–1.92)^∗^	1.15 (0.87–1.53)	1.80 (1.17–2.78)^∗^
Haemorrhagic stroke	1.34 (1.18–1.51)^∗∗∗^	2.17 (1.96–2.40)^∗^	1.18 (1.03–1.36)^∗^	1.73 (1.37–2.17)^∗^
Previous fracture	2.41 (2.34–2.48)^∗∗∗^	2.88 (2.72–3.04)^∗^	2.33 (2.20–2.48)^∗^	2.61 (2.33–2.93)^∗^

*Risk of fracture is presented as crude relative risk and 95% confidence intervals in exposed individuals as compared to non-exposed (controls). Each individual row represents the risk of fracture of one exposure variable (e.g., clopidogrel) when adjusted for the other exposures. CI, confidence interval; IRR, incidence rate ratio; ^∗^Two-tailed *P* < 0.05, ^∗∗^Two-tailed *P* < 0.01, ^∗∗∗^Two-tailed *P* < 0.001*.

**Table 3:** The risk of any fractures and of the three major groups of osteoporotic fractures (hip, forearm, and spine) is presented.

Crude relative risk	Any fracture	Hip fracture	Forearm fracture	Spine fracture
	HR (95% CI)	HR (95% CI)	HR (95% CI)	HR (95% CI)
Clopidogrel	0.85 (0.82–0.88)^∗^	0.65 (0.61–0.69)^∗^	0.90 (0.84–0.97)^∗^	0.77 (0.67–0.88)^∗^
TIA	1.05 (0.98–1.13)	1.13 (0.99–1.30)	1.08 (0.91–1.28)	1.09 (0.81–1.48)
Ischaemic stroke	1.14 (1.07–1.21)^∗^	1.02 (0.82–1.28)	0.98 (0.74–1.30)	1.12 (0.80–1.93)
Haemorrhagic stroke	1.04 (0.92–1.17)	1.19 (1.06–1.33)^∗^	0.97 (0.84–1.13)	1.07 (0.84–1.38)

*Risk of fracture is presented as crude relative risk, hazard ratio adjusted for multiple confounders. Each individual row represents the risk of fracture of one exposure variable (e.g., clopidogrel) when adjusted for the other exposures. Multiple confounders: Prior alcoholism, prior fractures, Charlson index, income, living alone or not, spironolactone use, use of bronchodilator drugs (proxy for smoking), use of drugs for smoking cessation (proxy for smoking), systemic corticosteroids, statin use, ACE use, ACE + diuretics use, combined alpha plus betablocker use, other diuretics use, betablocker use, betablocker plus other drug combined, calcium channel blocker, thiazide diuretics, loop diuretics, dipyridamole use, and acetylsalicylic use. HR, Hazard Ratio; CI, Confidence Interval; TIA, Transitory Ischemic Attack. ^∗^Two-tailed *P* < 0.05*.

As fracture risk is affected by gender, we stratified accordingly. Interestingly, increased risk of fractures was only seen in men, where it was significantly increased in men suffering ischaemic stroke (any fracture) and in men suffering haemorrhagic stroke (hip fracture) (**Table 4**).

**Table 4:** The risk of any fractures and of hip fractures stratified by gender is presented as crude relative risk, hazard ratio adjusted for multiple confounders.

Crude relative risk	Any fracture HR (95% CI)		Hip fracture HR (95% CI)	
	Men	Women	Men	Women
Clopidogrel	0.82 (0.78–0.87)^∗^	0.88 (0.84–0.93)^∗^	0.56 (0.51–0.62)^∗^	0.74 (0.68–0.81)^∗^
TIA	1.08 (0.98–1.20)	1.01 (0.92–1.10)	1.14 (0.92–1.40)	1.09 (0.91–1.30)
Ischaemic stroke	1.20 (1.09–1.31)^∗^	1.08 (0.99–1.17)	1.11 (0.82–1.51)	0.94 (0.69–1.29)
Haemorrhagic stroke	1.04 (0.87–1.23)	1.06 (0.89–1.25)	1.35 (1.14–1.59)^∗^	1.06 (0.91–1.23)

*Each individual row represents the risk of fracture of one exposure variable (e.g., clopidogrel) when adjusted for the other exposures. Multiple confounders: Prior alcoholism, prior fractures, Charlson index, income, living alone or not, spironolactone use, use of bronchodilator drugs (proxy for smoking), use of drugs for smoking cessation (proxy for smoking), systemic corticosteroids, statin use, ACE use, ACE + diuretics use, combined alpha plus betablocker use, other diuretics use, betablocker use, betablocker plus other drug combined, calcium channel blocker, thiazide diuretics, loop diuretics, dipyridamole use, and acetylsalicylic use. HR, Hazard Ratio; CI, Confidence Interval; TIA, Transitory Ischemic Attack. ^∗^Two-tailed *P* < 0.05*.

### Effect of Clopidogrel on Fracture Risk in Stroke Patients

**FIGURE 1:**
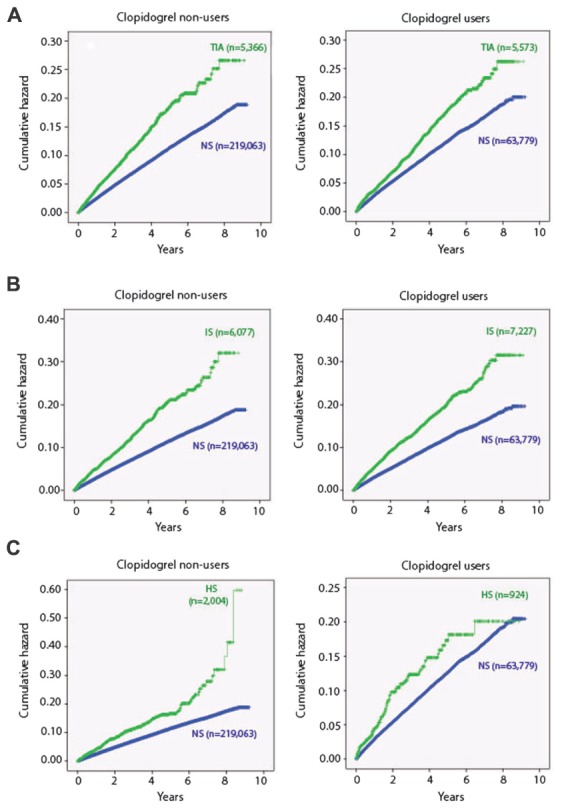
Cumulative hazard of any fracture as a function of time in years since index event. Both transitory ischemic attack (TIA) **(A)** and ischaemic stroke (IS) **(B)** increase the risk of any fracture and the risk continues to increase during the years after the event. Clopidogrel treatment does not seem to further increase the fracture. In patients with haemorrhagic stroke (HS) there is also an increased risk of fractures **(C)**. Again, clopidogrel does not affect the fracture risk until 6 years after the event. After 6 years there is a striking increase in risk in non-clopidogrel treated patients, while the risk in clopidogrel-users levels out and equals the risk in non-stroke subjects. NS: non-stroke/non-TIA.

We have previously shown that clopidogrel treatment increased fracture risk. Therefore, first we investigated whether clopidogrel treatment affected the fracture risk in the different categories of patients. In subjects with ischaemic stroke or TIA, no additional increase in fracture risk could be detected (**Figures 1A,B**). However, in subjects with haemorrhagic stroke, there was a striking change in fracture risk 6 years after the event. In clopidogrel-treated subjects, the risk leveled out and approached the risk in patients without stroke, while the risk in subjects not treated with clopidogrel increased dramatically (**Figure 1C**). Interestingly, when adjusting for multiple confounders clopidogrel treatment was associated with a reduced risk of all fracture types evaluated with a reduction in fracture risk between 10 and 35% (**Table 3**). Moreover, men had a more pronounced reduction in fracture risk than women for both any fracture (HR: 0.82 vs. 0.88, men and women, respectively) and for hip fracture (HR: 0.56 vs. 0.74) (**Table 4**).

As previous studies have shown a dose-dependent biphasic association between clopidogrel and fracture risk, we next stratified according to dose expressed as DDD/day. For all fracture types evaluated, the fracture risk was reduced by 10–46%, when DDD/day < 0.80. In contrast, for doses 0.8 DDD/day, fracture risk was either unaltered (any fracture and spine fractures), marginally reduced (hip fractures) or marginally increased (forearm fractures) when compared to individuals who never used clopidogrel (**Table 5**). Again, when stratifying for gender, the risk reducing effect was more pronounced in men than in women and for hip fractures than for any fracture (**Table 6**).

**Table 5:** The risk of any fractures and of the three major groups of osteoporotic fractures (hip, forearm, and spine) by dose of clopidogrel in all users.

Crude relative risk	Any fracture HR (95% CI)	Hip fracture HR (95% CI)	Forearm fracture HR (95% CI)	Spine fracture HR (95% CI)
Clopidogrel (never use as reference)				
<0.1 DDD/day	0.77 (0.74–0.81)^∗^	0.54 (0.48–0.60)^∗^	0.83 (0.74–0.92)^∗^	0.77 (0.63–0.94)^∗^
0.1–0.39 DDD/day	0.81 (0.760.85)^∗^	0.62 (0.55–0.69)^∗^	0.81 (0.72–0.91)^∗^	0.64 (0.50–0.80)^∗^
0.40–0.79 DDD/day	0.86 (0.80–0.92)^∗^	0.60 (0.52–0.70)^∗^	0.96 (0.83–1.12)	0.69 (0.51–0.94)^∗^
≥0.8 DDD/day	1.04 (0.99–1.11)	0.90 (0.81–1.00)^∗^	1.14 (1.00–1.29)^∗^	1.00 (0.79–1.25)
TIA	1.04 (0.97–1.11)	1.10 (0.96–1.26)	1.05 (0.89–1.25)	1.07 (0.79–1.44)
Ischaemic stroke	1.11 (1.04–1.18)^∗^	1.02 (0.82–1.27)	0.98 (0.74–1.30)	1.24 (0.80–1.92)
Haemorrhagic stroke	1.04 (0.92–1.17)	1.14 (1.02–1.28)^∗^	0.94 (0.81–1.10)	1.04 (0.81–1.34)

*Risk of fracture is presented as crude relative risk, hazard ratio adjusted for multiple confounders. Each individual row represents the risk of fracture of one exposure variable (e.g., clopidogrel) when adjusted for the other exposures. Multiple confounders: Prior alcoholism, prior fractures, Charlson index, income, living alone or not, spironolactone use, use of bronchodilator drugs (proxy for smoking), use of drugs for smoking cessation (proxy for smoking), systemic corticosteroids, statin use, ACE use, ACE + diuretics use, combined alpha plus betablocker use, other diuretics use, betablocker use, betablocker plus other drug combined, calcium channel blocker, thiazide diuretics, loop diuretics, dipyridamole use, and acetylsalicylic use. HR, Hazard Ratio; CI, Confidence Interval; TIA, Transitory Ischemic Attack. ^∗^Two-tailed *P* < 0.05*.

**Table 6:** The risk of any fractures and of the three major groups of osteoporotic fractures (hip, forearm, and spine) by dose of clopidogrel stratified by gender.

Crude relative risk	Any fracture HR (95% CI)		Hip fracture HR (95% CI)	
	Men	Women	Men	Women
Clopidogrel (never use as reference)
<0.1 DDD/day	0.78 (0.72–0.83)^∗^	0.80 (0.75–0.86)^∗^	0.49 (0.42–0.58)^∗^	0.61 (0.53–0.70)^∗^
0.1–0.39 DDD/day	0.81 (0.75–0.87)^∗^	0.86 (0.79–0.92)^∗^	0.52 (0.44–0.62)^∗^	0.76 (0.66–0.87)^∗^
0.40–0.79 DDD/day	0.81 (0.73–0.90)^∗^	0.93 (0.84–1.03)	0.52 (0.41–0.66)^∗^	0.70 (0.58–0.85)^∗^
≥0.8 DDD/day	0.97 (0.89–1.07)	1.01 (0.93–1.08)	0.79 (0.66–0.94)^∗^	0.91 (0.80–1.04)
TIA	1.07 (0.97–1.19)	0.99 (0.91–1.09)	1.11 (0.90–1.36)	1.06 (0.89–1.27)
Ischaemic stroke	1.17 (1.07–1.29)^∗^	1.05 (0.96–1.15)	1.11 (0.82–1.51)	0.94 (0.69–1.29)
Haemorrhagic stroke	1.03 (0.87–1.23)	1.06 (0.89–1.25)	1.31 (1.11–1.54)^∗^	1.02 (0.88–1.19)

*Risk of fracture is presented as crude relative risk, hazard ratio adjusted for multiple confounders. Each individual row represents the risk of fracture of one exposure variable (e.g., clopidogrel) when adjusted for the other exposures. Multiple confounders: Prior alcoholism, prior fractures, Charlson index, income, living alone or not, spironolactone use, use of bronchodilator drugs (proxy for smoking), use of drugs for smoking cessation (proxy for smoking), systemic corticosteroids, statin use, ACE use, ACE + diuretics use, combined alpha plus betablocker use, other diuretics use, betablocker use, betablocker plus other drug combined, calcium channel blocker, thiazide diuretics, loop diuretics, dipyridamole use, and acetylsalicylic use. HR, Hazard Ratio; CI, Confidence Interval; TIA, Transitory Ischemic Attack. ^∗^Two-tailed *P* < 0.05*.

## Discussion

In the present study, we observed a clear association between occurrence of stroke and risk of osteoporotic fractures. Moreover, we found that patients treated with the P2Y12 inhibitor, clopidogrel, overall had minimally affected fracture risk when compared to non-users. However, when stratifying for dosage and duration expressed as DDD/day, we found that the fracture risk was reduced by as much as 50% for less adherent patients while adherent patients treated with the recommended dose had the same fracture risk as non-users.

It is well known that immobilization following a stroke leads to increased bone resorption and decreased bone formation (Sato et al., 2000) and bone loss (Ramnemark et al., 1999). Excessive bone loss is a clear risk factor of osteoporosis and subsequently fractures. Also, stroke patients have a significantly increased incidence of falls, which aggravates the risk of osteoporotic fractures in these patients. A recent meta-analysis included 13 cohorts of stroke survivors and found a 50% increased risk of hip fracture in the stroke patients compared to men and women of the same age (Yuan et al., 2016). This corresponds well with our findings, where we see a 50% increase in risk in both any fracture and hip fracture in patients with ischaemic stroke, while the risk of spine fractures increase by as much as 80%. The increase in fracture risk is even higher in patients with haemorrhagic stroke, where risk of hip fractures is increased by as much as 120%. Hip fractures are especially serious in stroke patients, as hip fractures are associated with a high mortality, disability, loss of independence, and high medical costs.

Interestingly TIA was also associated with an increase in fracture risk of between 18 and 50%, but after adjusting for multiple confounders no increased fracture risk could be detected. In these patients, symptoms of the ischaemic attack would disappear within 24 h. However, TIA patients may have underlying common risk factors for fracture and stroke, or they might have minor, undetected neurological deficits after the TIA that affects balance and increases fall incidence as well as they have an increased risk of developing more severe stroke at a later time point the clinician should still evaluate the fracture risk and bone health of these patients.

To our knowledge, this is the first study to investigate the association between fracture risk and clopidogrel use in stroke and TIA patients. It is particularly important to elucidate the effect of clopidogrel in stroke patients. First, they are at increased risk of osteoporosis and subsequent fractures due to immobilization and fall tendency and they are more vulnerable than other patient. Next, we have previously shown that clopidogrel treatment is associated with an increased risk of fracture in a nationwide cohort study including all patients prescribed clopidogrel Denmark (Jørgensen et al., 2012). Finally, we found that the patients with the highest risk where those receiving long-term treatment (more than 1 year) and as clopidogrel treatment in stroke patients is life-long, it is highly relevant to address the association of clopidogrel treatment on fracture risk in this particular patient category. In contrast to our previous study that included patients prescribed clopidogrel for any indication, we only find a marginally increased risk of osteoporotic fractures, and after adjusting for multiple confounders known to predispose to osteoporosis and fractures, we actually find a reduced risk of all fracture types. This is even more pronounced when we stratify for dose, where less adherent patients have even more reduced fracture risk as compared to both non-users and adherent patients receiving recommended doses. The obvious explanation for this would be that clopidogrel, at least in lower doses or shorter duration, affects bone metabolism and protects against fractures. This is supported by an *in vivo* study in which P2Y12 null mice had reduced osteoclastic activity and were partially protected against pathological bone loss (Su et al., 2012). However, another *in vivo* study from our own group found that clopidogrel treatment of mice induced bone loss and reduced bone strength (Syberg et al., 2012), so the effects of clopidogrel on bone cells and bone metabolism are not fully elucidated yet. Another explanation for the reduced fracture risk in less adherent patients could be that these patients stop the treatment due to side effects or they could be more severely affected by the stroke and are thus completely immobilized and thus in bed for prolonged periods of time, thus reducing the risk of falls and subsequent fractures.

As fracture risk differs between men and women we stratified the analyses according to gender. Interestingly, we found that clopidogrel treatment in men is associated with a lower fracture risk than in women. It has previously been shown that the effects of clopidogrel on platelet function are gender dependent (Hobson et al., 2009; Patti et al., 2014), and our data suggests that this might be a more universal gender-specific effect of clopidogrel in all target organs. However, differences in underlying comorbidities could also lead to over- or underadjustment for confounders in the analyses, falsely demonstrating an effect of gender on the response to clopidogrel.

Due to the centrally organized healthcare system in Denmark, a broad range of healthcare information is collected and can be collated based on the unique identification number for each citizen in Denmark. This enabled us to perform a large-scale population-based designed study. This also made it possible to collect data on confounders and exposure before the occurrence of fracture, thereby eliminating the risk of recall bias. Moreover, as all collected prescriptions are registered with the Danish Medicines Agency, it has been possible to include all patients prescribed clopidogrel in the study, thereby reducing the risk of selection bias. Finally, as almost all fractures are treated in public emergency departments and hospitals covered by the databases, collection of fracture data is considered almost complete, though there can be a minor risk of coding errors by the discharging physicians.

Though the strength of the study is the large-scale design, the optimal design would have been a prospective, randomized trial as optimal confounder-control could have been obtained. Such study should have included the determination of circulating markers of bone turnover to determine the effects of clopidogrel on bone formation and bone resorption in the patients. However, it would not have been feasible to conduct such a trial, as the cost of a sufficiently powered study with fracture as endpoint would have been immense. Moreover, it is not ethically justifiable to conduct a placebo-controlled trial, as clopidogrel is part of the standard, recommended treatment after an ischaemic stroke as well as after other cardiovascular diseases.

In our study, we have extensively controlled for multiple confounders in relation to fractures, but as in all observational studies there might still be residual confounding because of possible differences in comorbidities. Especially, the choice of prescribing clopidogrel to the stroke patient is dependent on the type of stroke (haemorrhagic, ischaemic) and possibly also the general condition of the patient. Thus, the severity of neurological deficit after the cerebrovascular event might be different between the treatment groups, which we are not able to control for with the current design. Also, differences in extent of exercise and mobilization and thus the risk of falls has not been possible to adjust for, as this information is not contained in any registers.

Future studies should address the effects of the newer P2Y12 receptor antagonists, prasugrel and ticagrelor, as they have other modes of function on the receptor. However, as they were marketed later than clopidogrel and have not been implemented as part of the standard of care in the treatment of stroke in Denmark, the number of patients treated would not be sufficient and the study therefore not sufficiently powered to determine the effects on osteoporotic fractures.

## Conclusion

Patients with stroke have increased risk of osteoporotic fractures, but treatment with the widely used platelet inhibitor, clopidogrel, does not seem to increase the risk of fractures. In contrast, less adherent patients have lower risk of fractures than non-users. This might be due to a bone-protective effect of clopidogrel at lower doses or it might be due to differences in comorbidities between patients exposed to suboptimal doses of clopidogrel and patients treated with recommended doses. Thus, clopidogrel is not associated with deleterious effects on bone in stroke patients, but might even reduce the risk of fracture in these patients. However, further studies should investigate the exact effects of clopidogrel on bone metabolism.

Finally, in light of the high risk of fractures in stroke patient *per se* and the large number of underlying risk factors, careful attention should be given to the bone status of these patients. Clinicians should carefully consider additional evaluation of the bone status of stroke survivors and treatment should be given to prevent development or progression of osteoporosis.

## Author Contributions

Conception and design of the study: NJ, PV, HI, and PS. Acquisition, analysis, and interpretation of data: NJ, PV, HI, and PS. Drafting of the work: NJ and PV. Revising it critically for intellectual content: NJ, PV, HI, and PS. Final approval of the version to be published: NJ, PV, HI, and PS. Agreement to be accountable for all aspects of the work in ensuring that questions related to the accuracy or integrity of any part of the work are appropriately investigated and resolved: NJ, PV, HI, and PS.

## Conflict of Interest Statement

The authors declare that the research was conducted in the absence of any commercial or financial relationships that could be construed as a potential conflict of interest.

## Footnotes

1 http://www.dkma.dk
